# Supplementary material for: Label-free prediction of cell painting from brightfield images
Source: Sci Rep. 2022 Jun 15;12:10001. doi: 10.1038/s41598-022-12914-x (PMC9200748; doi:10.1038/s41598-022-12914-x)
Supplement: Supplementary file 1 — Supplementary Information 1. [file 41598_2022_12914_MOESM1_ESM.docx]

**Supplementary Information**


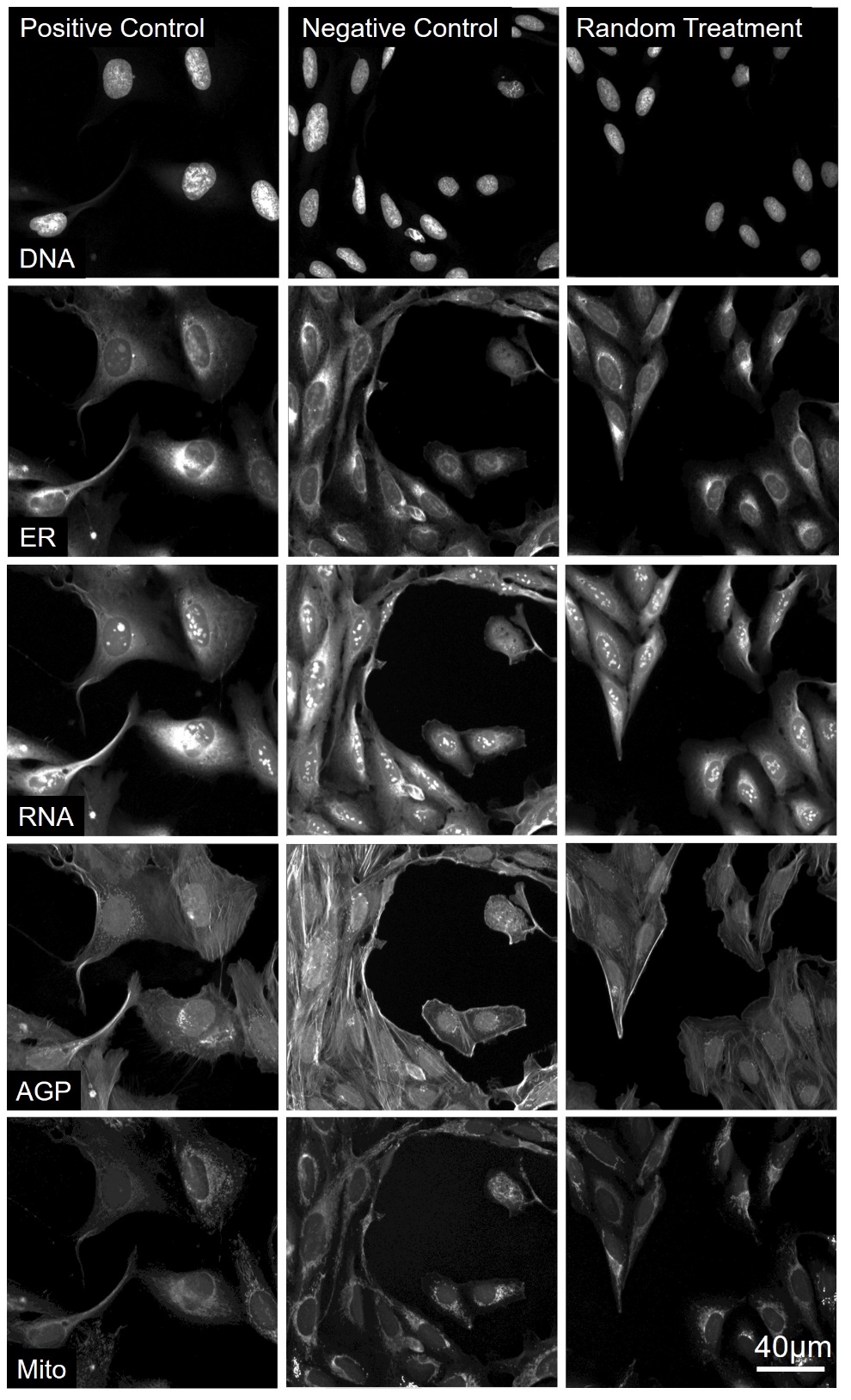
**SUPP Figure A Legend:** Typical examples of the three compound treatment groups from our dataset: positive control, negative control and random treatment. Five channels are displayed for each example to highlight visual differences between the treated cells, notably the increased size and sparsity of the cells in the positive control group.


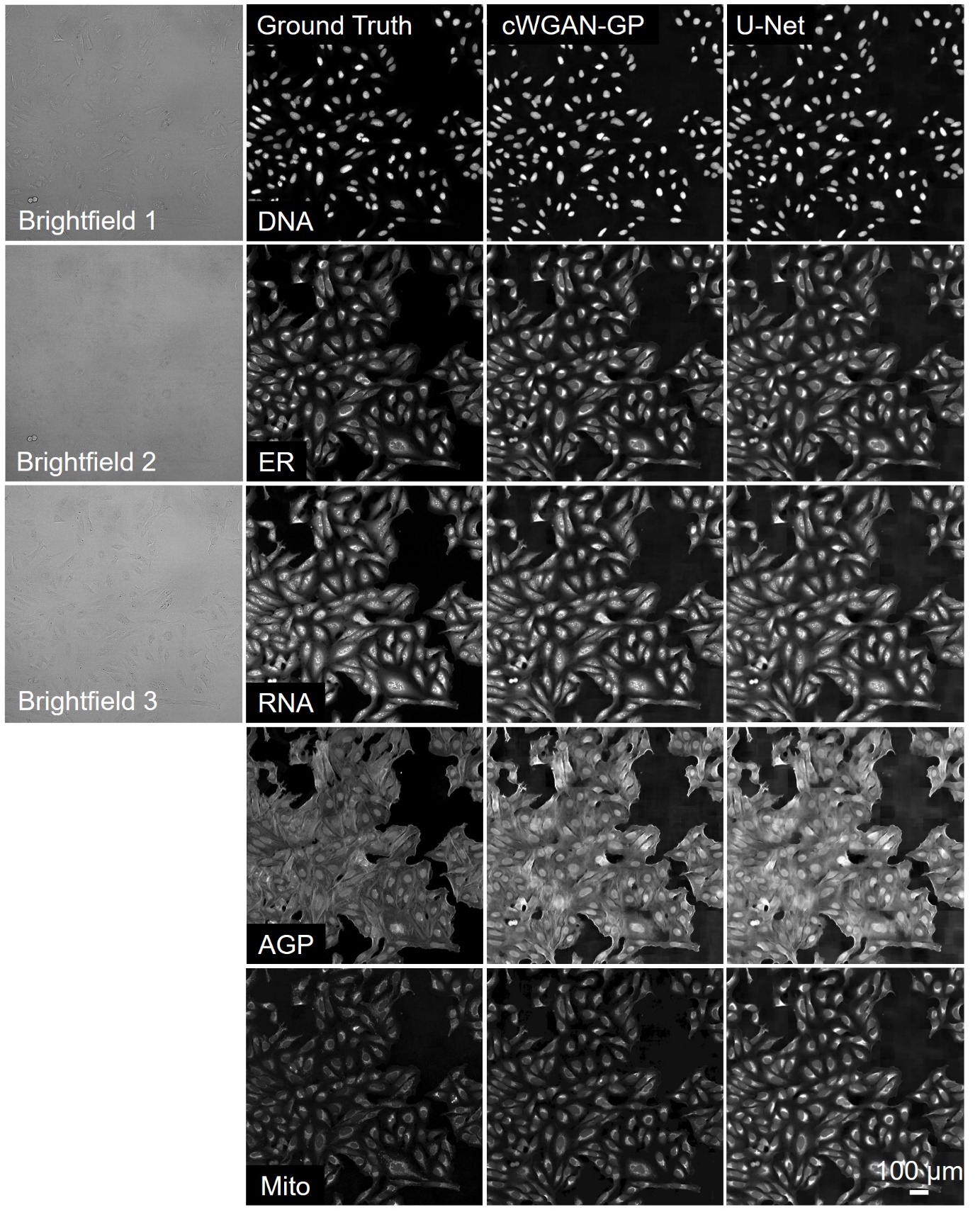
**SUPP Figure B Legend:** A typical example of brightfield, ground truth fluorescent, and predicted channels from the test dataset for the U-Net and cWGAN-GP models. The images are as they are used in the CellProfiler analysis (998 x 998 pixels), representing a full field of view. Images are independently contrasted for visualization

**
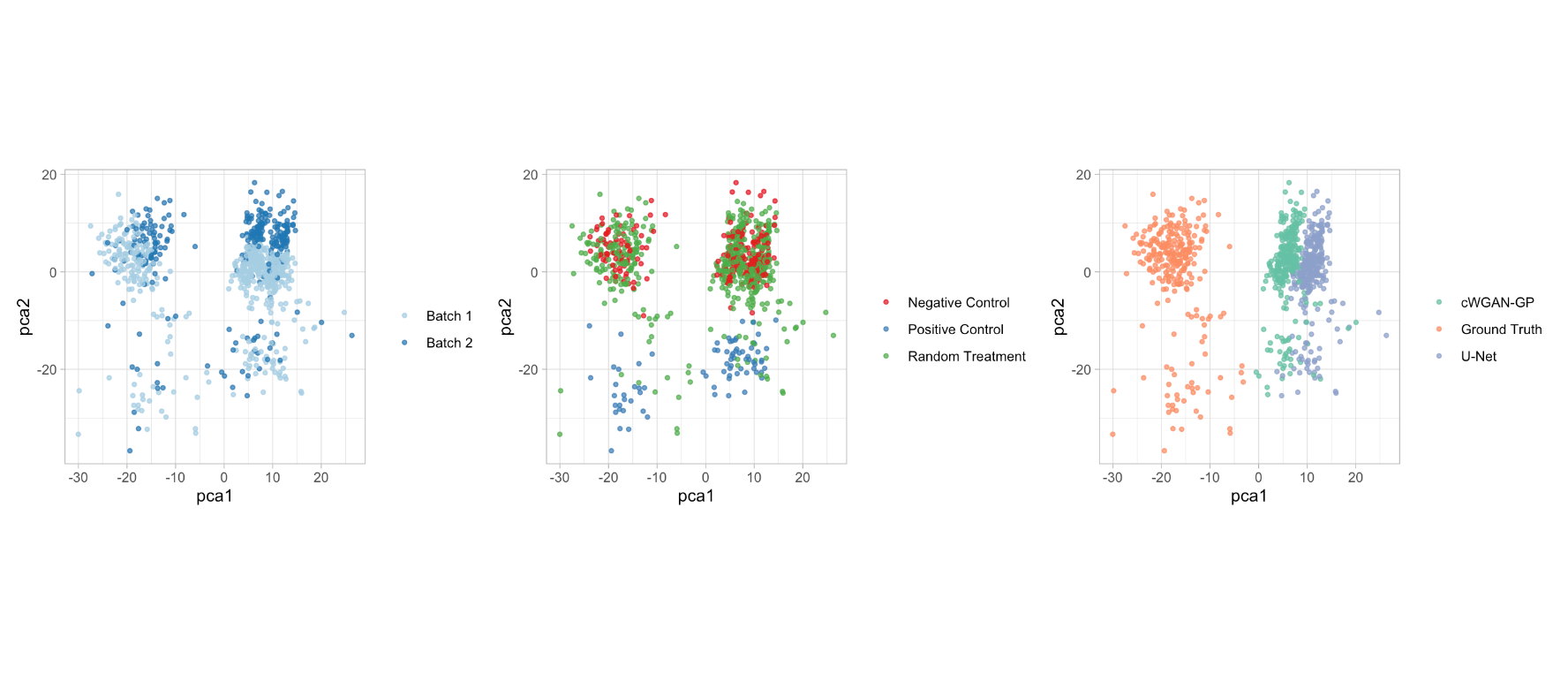
SUPP Figure C Legend:** PCA is used to replicate the UMAP results, showing that that both models can reproduce the separation between treatments and batches seen in the ground truth features. cWGAN-GP lies closer to the ground truth than U-Net, although the two models sit much closer to each other in feature space than to the ground truth. A full-sized version of this image is available in the “Supplementary” folder of our GitHub repository (<https://github.com/crosszamirski/Label-free-prediction-of-Cell-Painting-from-brightfield-images>).

**SUPP Figure D Legend:** Density plots of feature correlation to ground truth for both models (U-Net and cWGAN-GP) by Feature Site and Feature Type (for all features after feature selection).

**
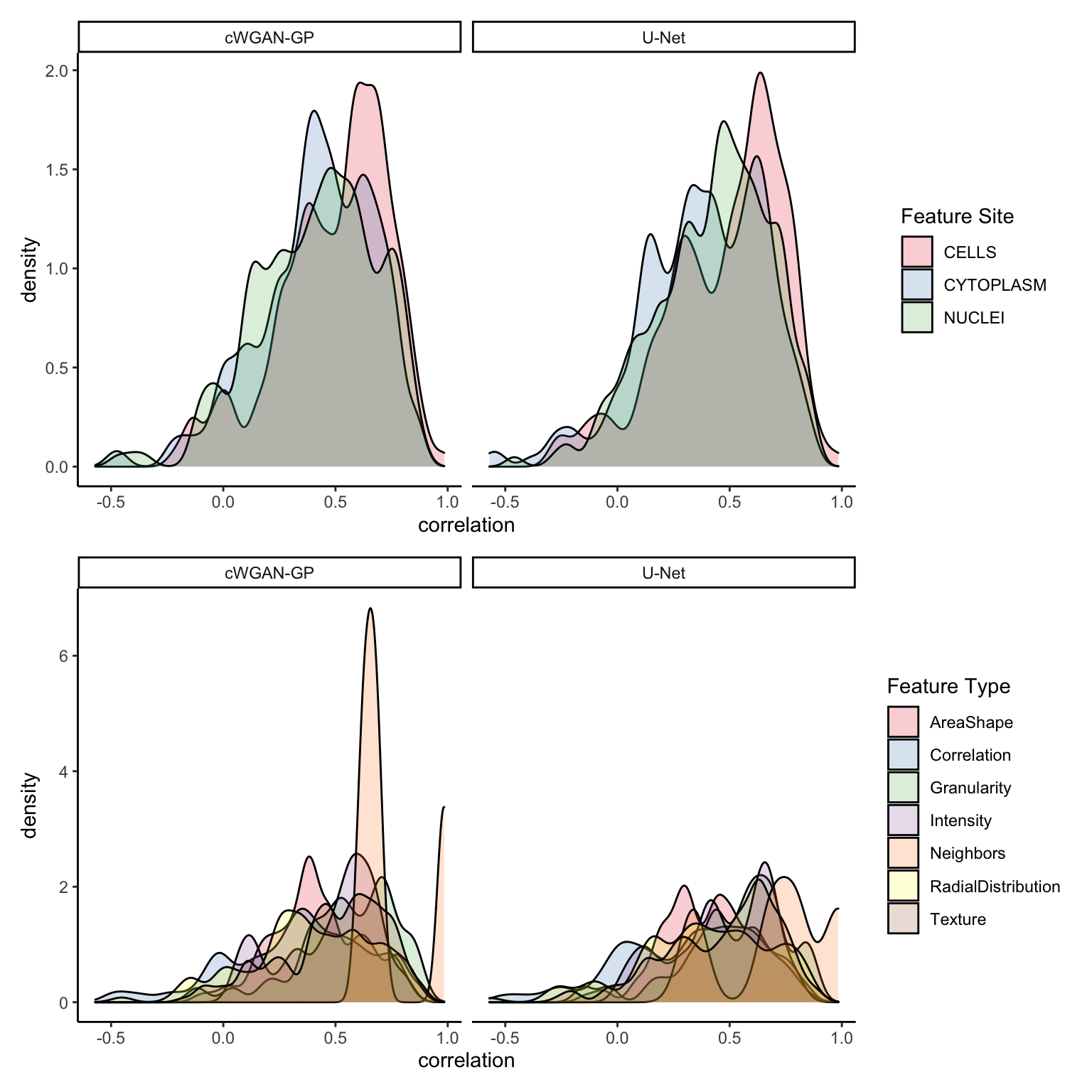
**

**SUPP Figure E Legend:** Venn diagram of K-NN classifier results representing the number of compounds identified as clustering with the feature profile of positive control compound mitoxantrone. Four distinct compounds were identified by all three methodologies, including clinical-stage compounds glipizide and GW-842470 (structures shown).


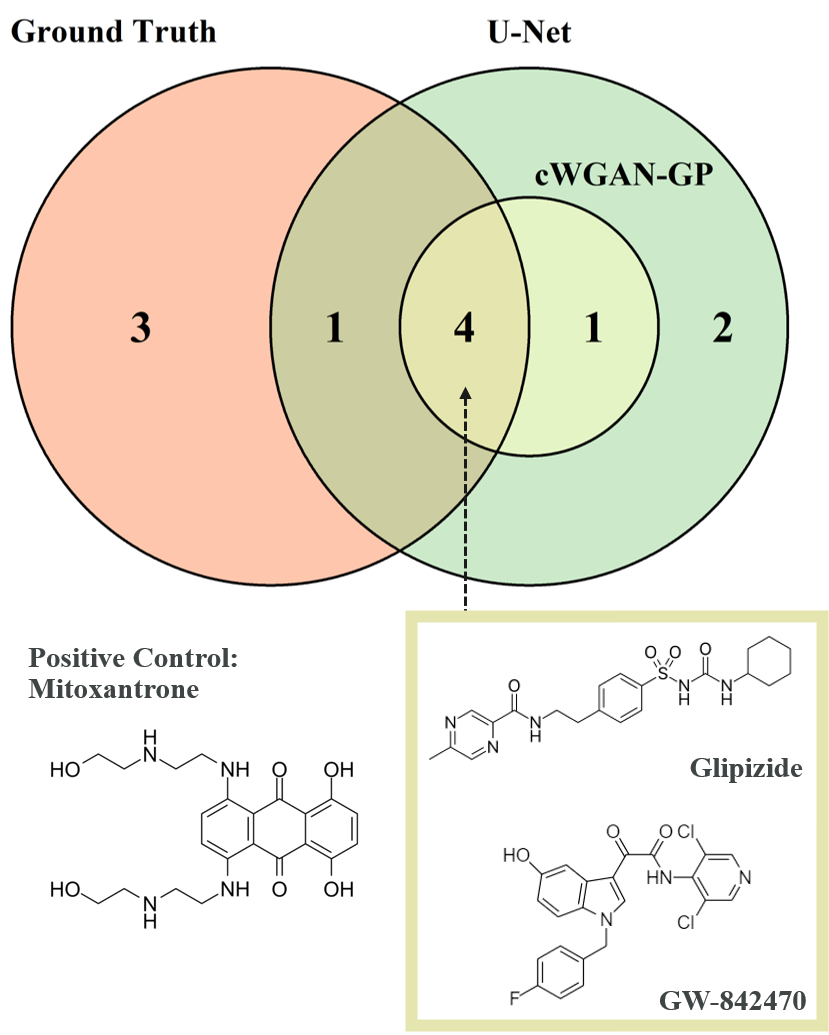


**SUPP Figure F Legend:** cWGAN-GP extracted features ranked by correlation to ground truth by feature site and feature type. Raw data in Supplementary Table C. A full-sized version of this image is available in the “Supplementary” folder of our GitHub repository.


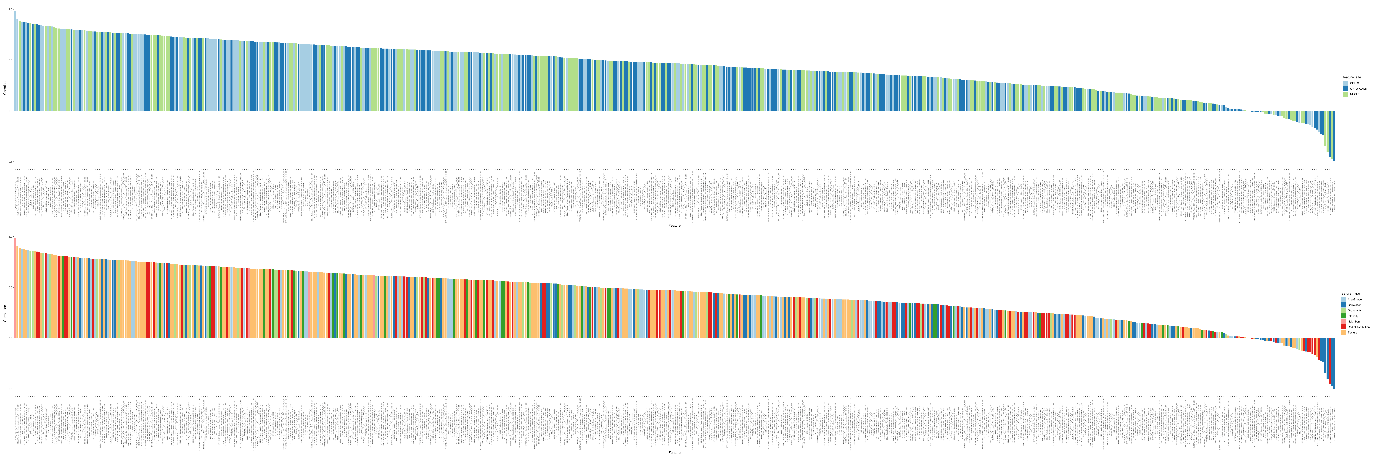


**SUPP Figure G Legend:** U-Net extracted features ranked by correlation to ground truth by feature site and feature type. Raw data in Supplementary Table D. A full-sized version of this image is available in the “Supplementary” folder of our GitHub repository.


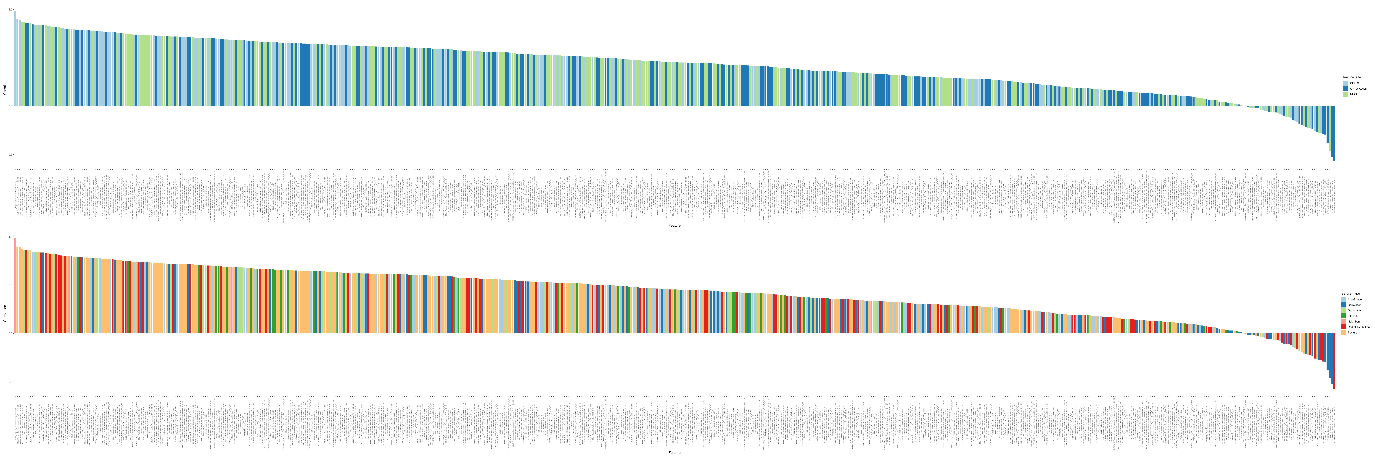


**SUPP Table A Legend:** Cell Painting stain preparation details by channel for our dataset.

| **Imaging Channel** | **Stain** | **Stock Preparation** | **Stock Concentration** | **Dilution Factor** | **Working Concentration** |
| --- | --- | --- | --- | --- | --- |
| **DNA** | Hoechst 33342 (Thermo #H3570) | Use as provided | 10 mg/mL | 1:2000 | 5 µg/mL |
| **ER** | Concanavalin A / Alexa Fluor 488 (Thermo #C11252) | Add 1 mL 0.1M sodium bicarbonate (in dH_2_O) to vial | 5 mg/mL | 1:500 | 10 µg/mL |
| **RNA** | SYTO 14 green fluorescent nucleic acid stain (Thermo #S7576) | Use as provided | 5 mM | 1:555.5 | 9 µM |
| **AGP** | Wheat-germ agglutinin / Alexa Fluor 555  (Thermo #W32464) | Add 5 mL dH2O to vial, centrifuge at 10’000g for 30s to remove aggregates | 1 mg/mL | 1:666.7 | 1.5 µg/mL |
|  | Phalloidin / Alexa Fluor 568 (Thermo #A12380) | Add 1.5 mL 100% (v/v) methanol to vial | 1 mL/mL  (300 units in vial) | 1:200 | 5 µL/mL  (1.5 units) |
| **Mito** | MitoTracker Deep Red  (Thermo #M22426) | Add 91 µL DMSO to vial | 1 mM | 1:2000 | 0.5 µM |

**SUPP Table B Legend:** Full CellProfiler feature list prior to feature reduction in .csv format.

**SUPP Table C Legend:** cWGAN-GP raw correlation data in .csv format.

**SUPP Table D Legend:** U-Net raw correlation data in .csv format.
