# Supplementary figures and images for: Label-free prediction of cell painting from brightfield images
Source: Sci Rep. 2022 Jun 15;12:10001. doi: 10.1038/s41598-022-12914-x (PMC9200748; doi:10.1038/s41598-022-12914-x)

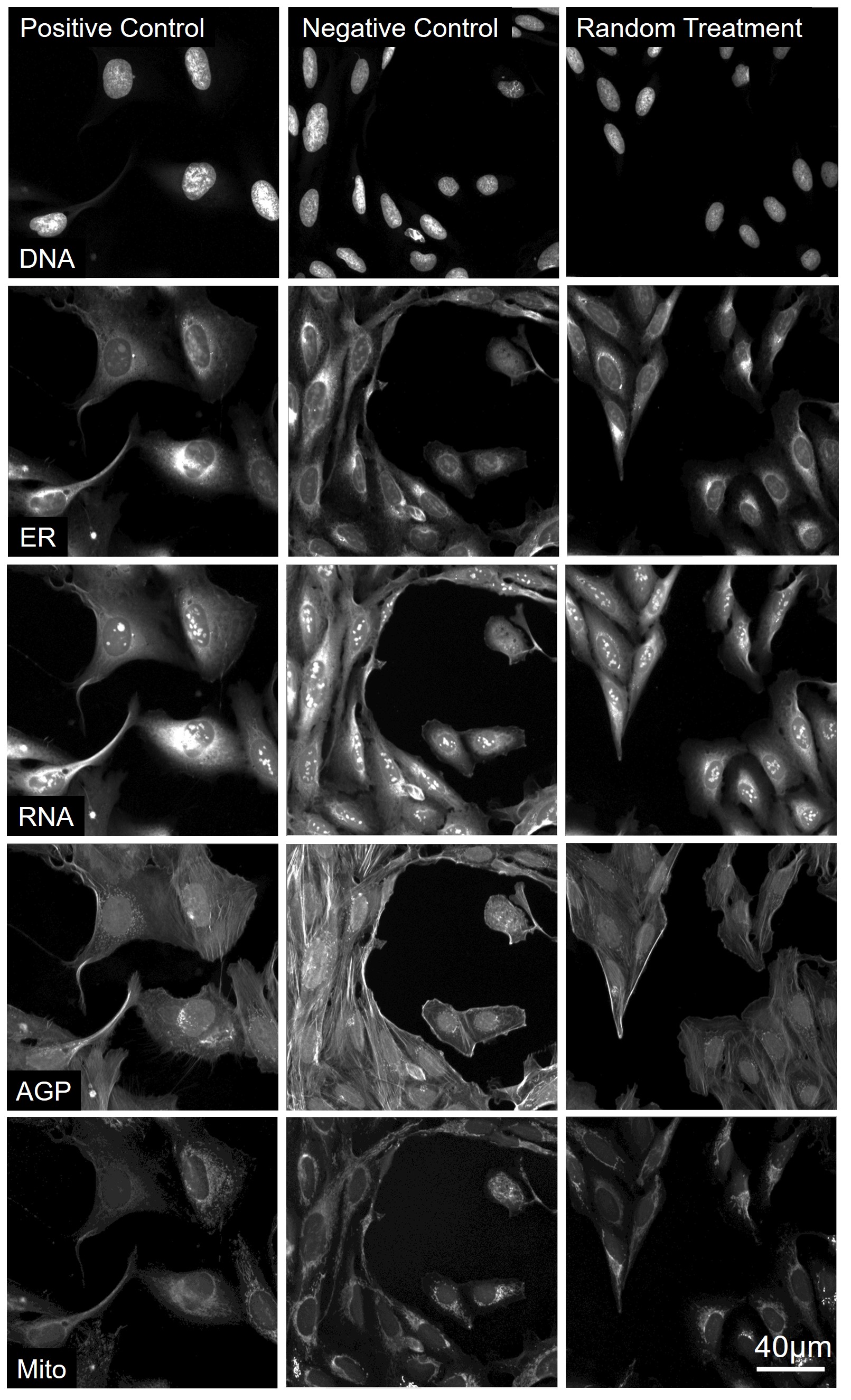

Supplement: Supplementary file 2 — Supplementary Information 2. [file 41598_2022_12914_MOESM2_ESM.jpg]

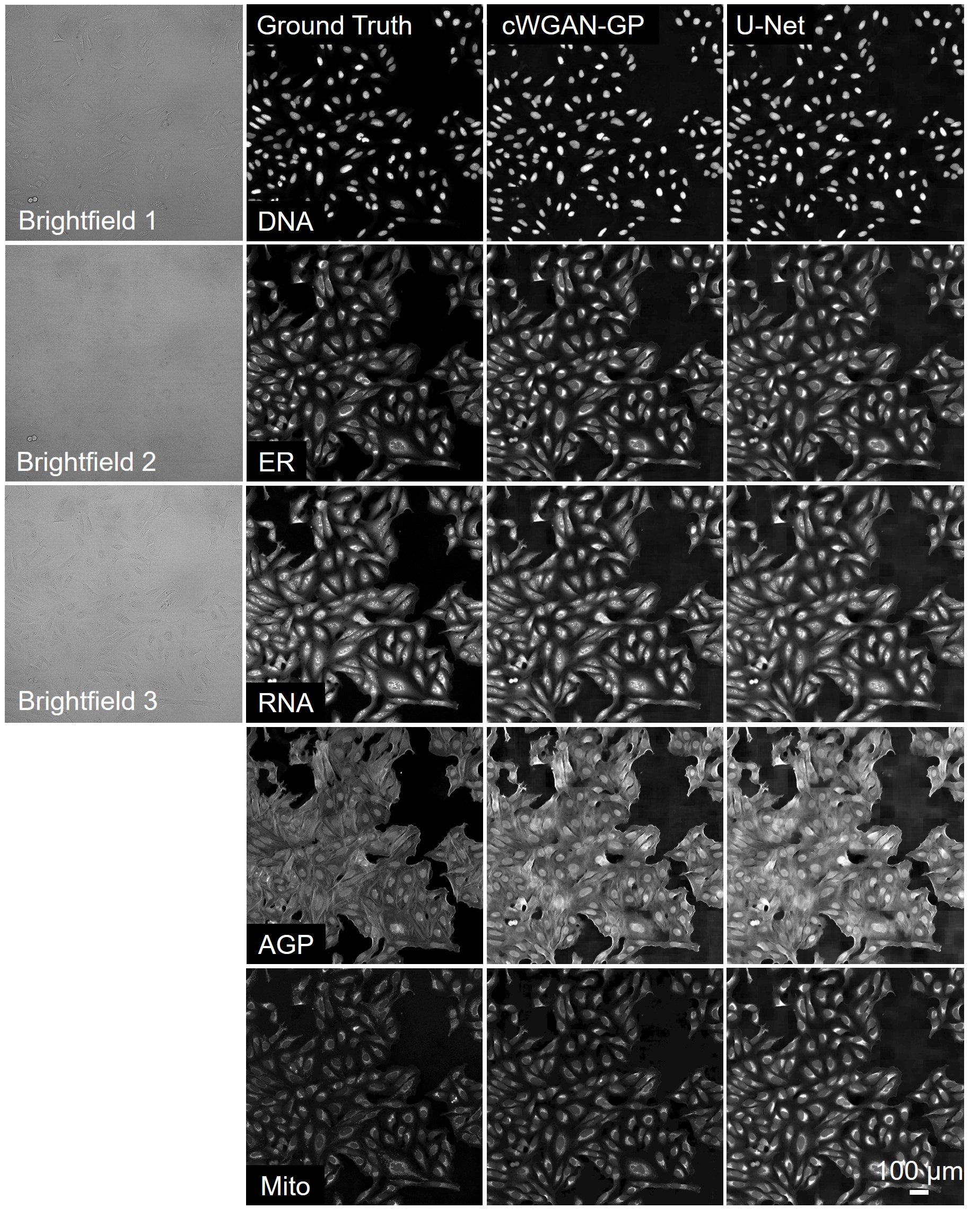

Supplement: Supplementary file 3 — Supplementary Information 3. [file 41598_2022_12914_MOESM3_ESM.jpg]

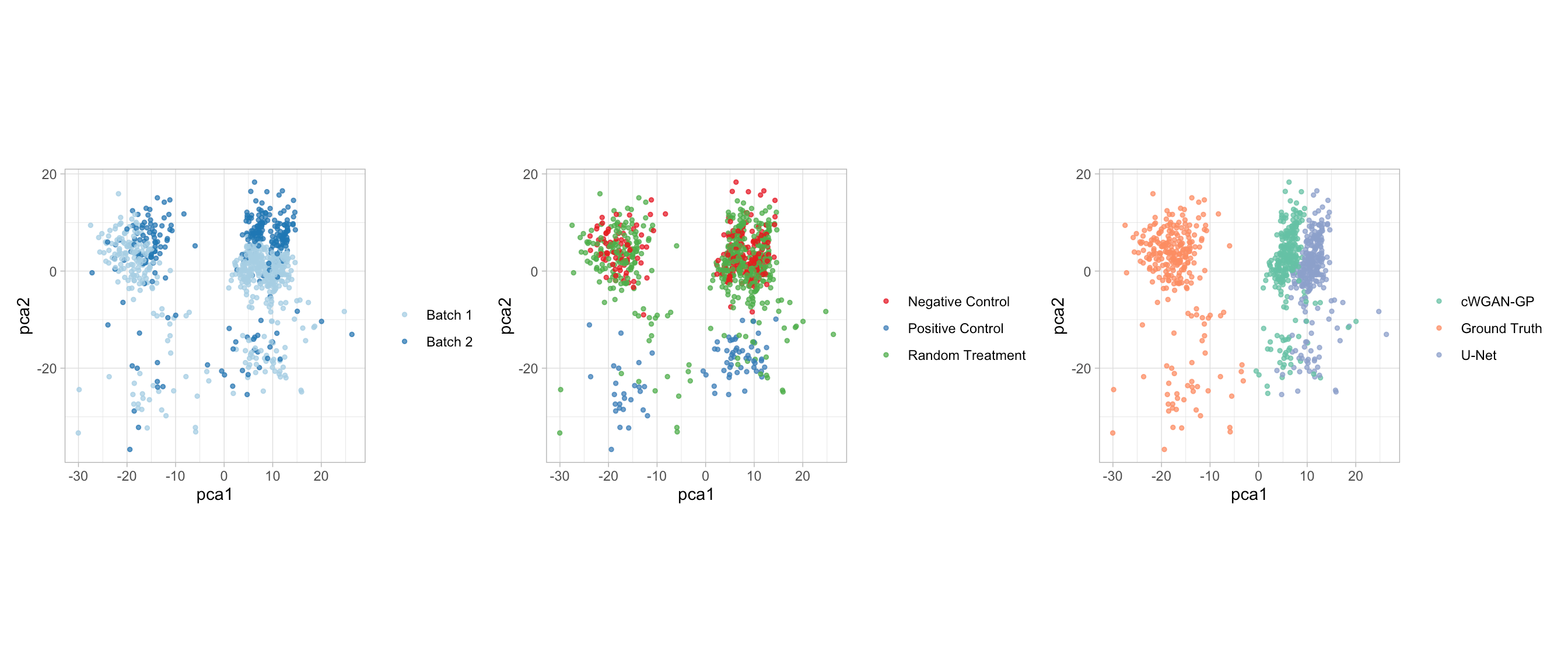

Supplement: Supplementary file 4 — Supplementary Information 4. [file 41598_2022_12914_MOESM4_ESM.png]

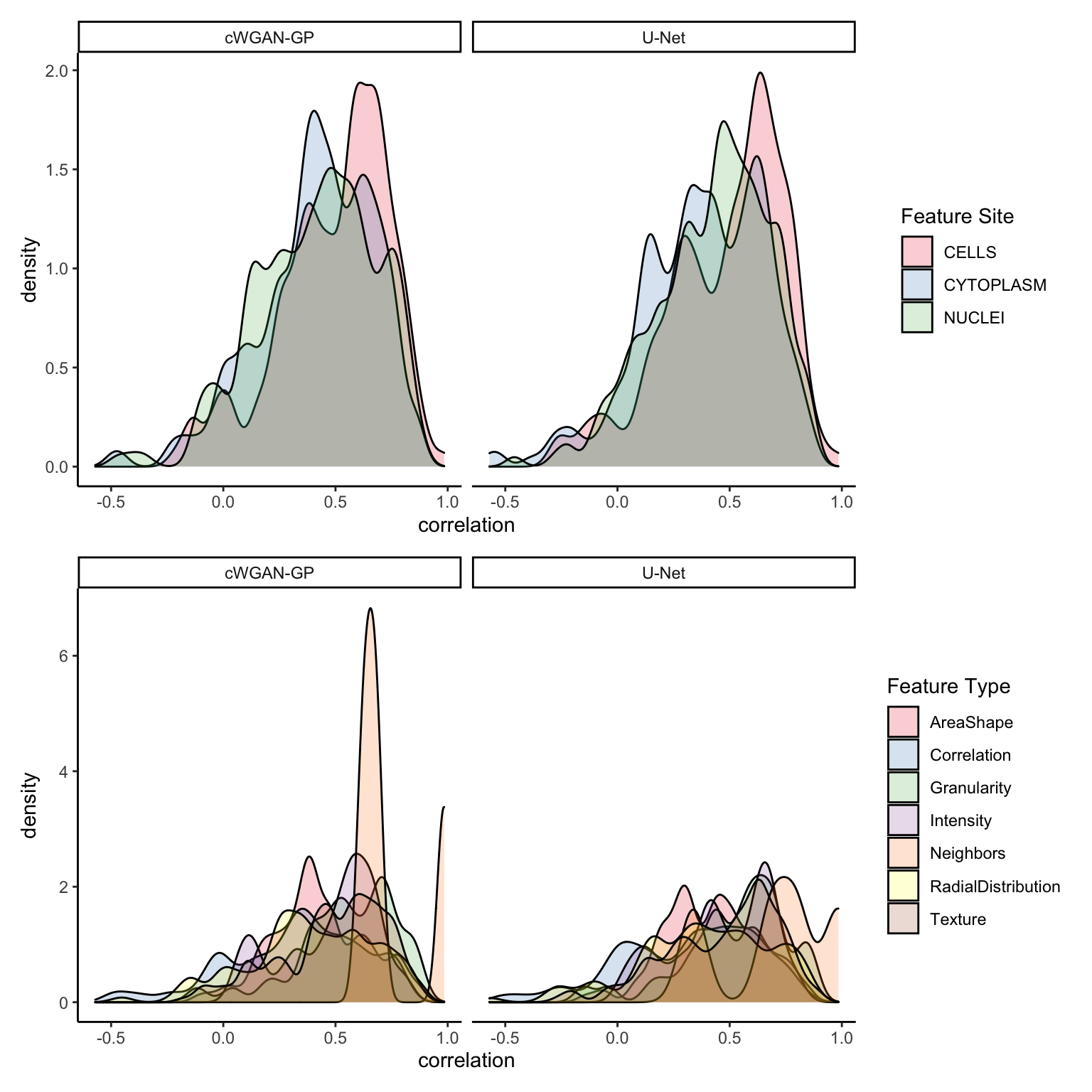

Supplement: Supplementary file 5 — Supplementary Information 5. [file 41598_2022_12914_MOESM5_ESM.png]

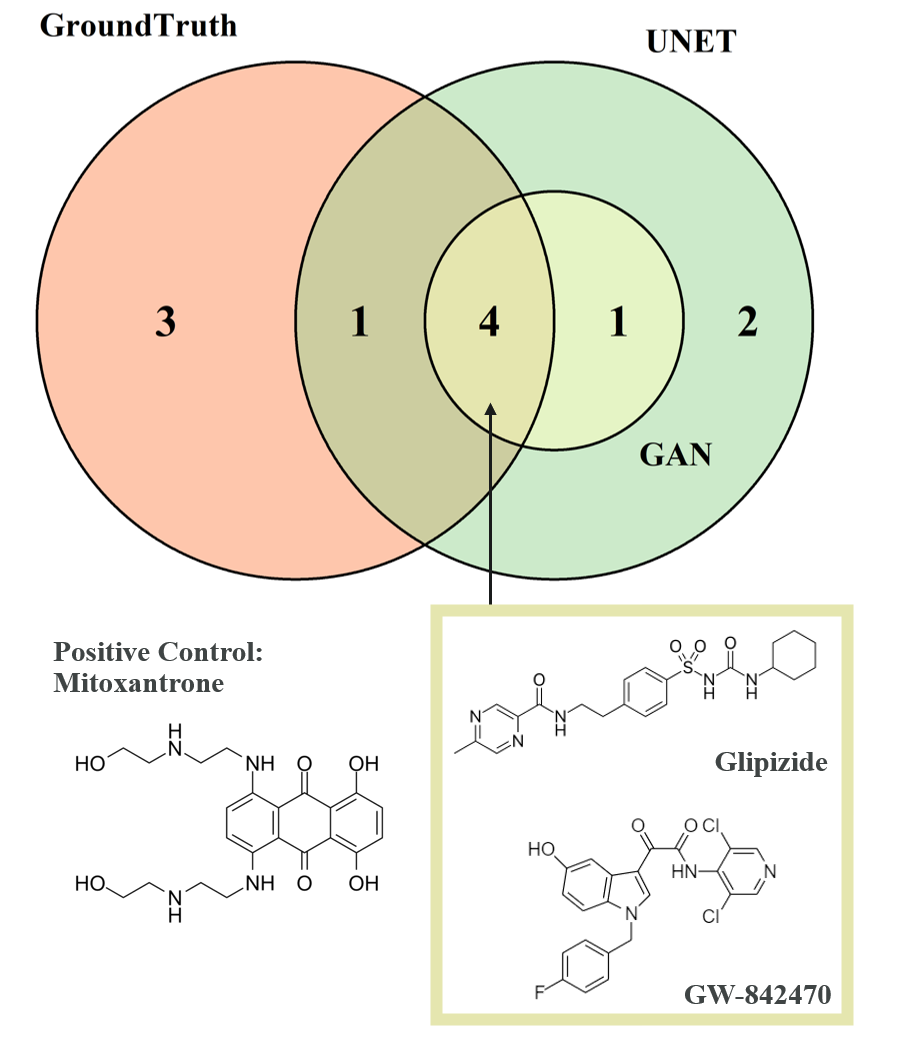

Supplement: Supplementary file 6 — Supplementary Information 6. [file 41598_2022_12914_MOESM6_ESM.png]

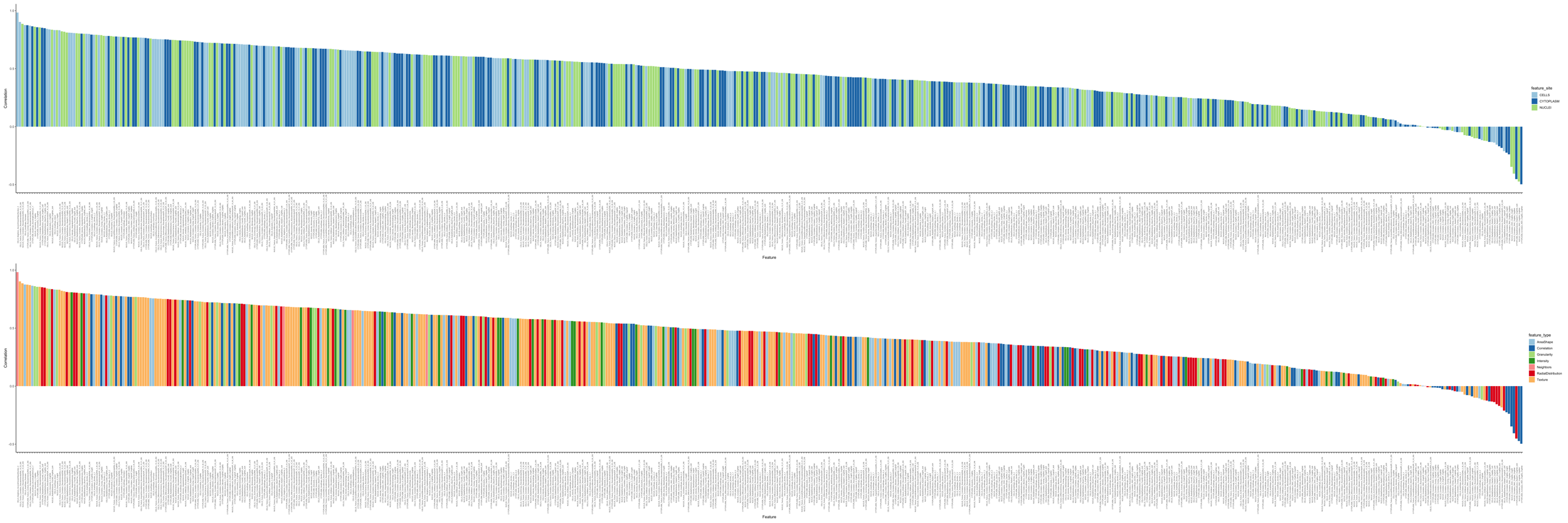

Supplement: Supplementary file 7 — Supplementary Information 7. [file 41598_2022_12914_MOESM7_ESM.png]

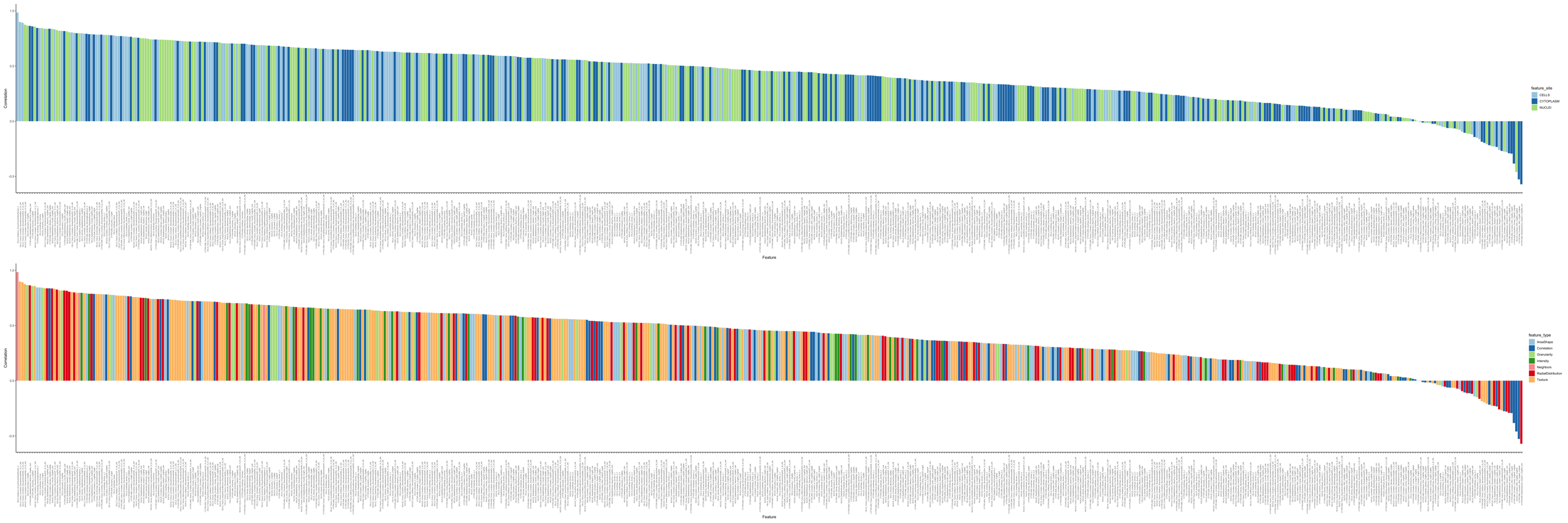

Supplement: Supplementary file 8 — Supplementary Information 8. [file 41598_2022_12914_MOESM8_ESM.png]
